# Machine learning applications in drug development

**DOI:** 10.1016/j.csbj.2019.12.006

**Published:** 2019-12-26

**Authors:** Clémence Réda, Emilie Kaufmann, Andrée Delahaye-Duriez

**Affiliations:** a NeuroDiderot, UMR 1141, Inserm, Université de Paris, Sorbonne Paris Cité, Hôpital Robert Debré, 48, boulevard Sérurier, Paris 75019, France; b Université Paris Diderot, Université de Paris, Sorbonne Paris Cité, 5, rue Thomas Mann, Paris 75013, France; c Univ. Lille, CNRS, Centrale Lille, Inria, UMR 9189 - CRIStAL - Centre de Recherche en Informatique Signal et Automatique de Lille, F-59000 Lille, France; d Université Paris 13, Sorbonne Paris Cité, UFR de santé, médecine et biologie humaine, Bobigny 93000, France; e Service histologie-embryologie-cytogénétique-biologie de la reproduction-CECOS, Hôpital Jean Verdier, AP-HP, Bondy 93140, France

**Keywords:** Drug discovery, Drug repurposing, Multi-armed bandit, Collaborative filtering, Bayesian optimization, Adaptive clinical trial

## Abstract

Due to the huge amount of biological and medical data available today, along with well-established machine learning algorithms, the design of largely automated drug development pipelines can now be envisioned. These pipelines may guide, or speed up, drug discovery; provide a better understanding of diseases and associated biological phenomena; help planning preclinical wet-lab experiments, and even future clinical trials. This automation of the drug development process might be key to the current issue of low productivity rate that pharmaceutical companies currently face. In this survey, we will particularly focus on two classes of methods: sequential learning and recommender systems, which are active biomedical fields of research.

## Highlights

• Applications of sequential learning and recommender systems to pharmaceutics.
• Review of Machine Learning methods in drug discovery, testing and repurposing.
• Survey of available genomic data and feature selection methods for drug development.

## Graphical abstract

**Figure:**
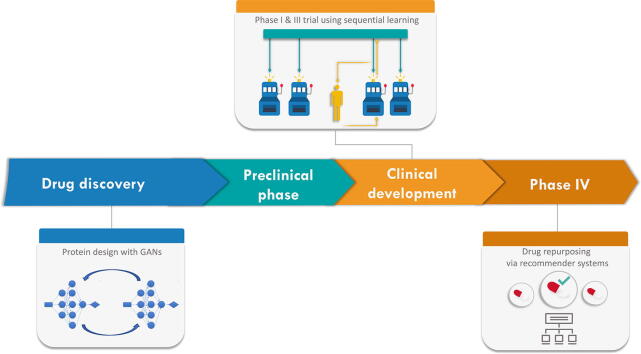

## 1 Introduction

A great variety of experimental data, at a chemical, transcriptomic, or genomic-level is available to readily use for drug development. Summarizing the huge amount of biological data at hand into meaningful models, to grasp the full mechanism of diseases, seems harder and harder. However, systems biology and machine learning approaches are continuously enhanced in order to accelerate the path to efficient drug development. We will focus on three significant related and intermingled questions, that can be subject to automation: drug discovery, drug testing, and drug repurposing. Firstly, this review briefly dwells on the current context in drug development. Later, we will review generic machine learning algorithms, and more specifically, we will focus on sequential learning algorithms and recommender systems. These algorithms have also proven themselves useful in other research fields, and are active biomedical fields of research.

### 1.1 Drug development

#### 1.1.1 Current context in drug development

**Fig. 1:**
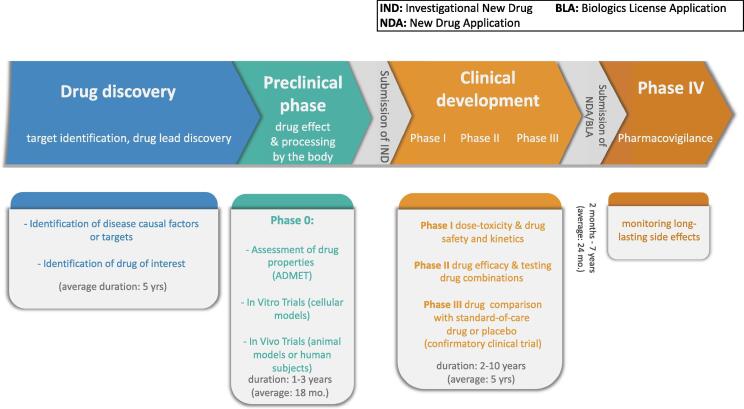
Representation of the four stages of drug development, along with Phase IV, which occurs after the start of drug marketing.

Development of new drugs is a time-consuming and costly process. Indeed, in order to ensure both the patients' safety and drug effectiveness, prospective drugs must undergo a competitive and long procedure. Drug development is roughly split into four major stages, called phases. Phase 0 comprises basic research/drug discovery and preclinical tests, which aim at assessing the efficiency and body processing of the drug candidate. The last three stages are clinical trials: study of dose-toxicity, short-lived side effects, and kinetic relationships (Phase I); determination of drug performance (Phase II); and comparison of the molecule to the standard-of-care (Phase III). An optional Phase IV can be post-drug marketing to monitor long-lasting side effects and drug combination with other therapies. See Fig. 1 for the whole drug development timeline. This pipeline takes at least 5 years to be completed [1], and can last up to 15 years [2]. The minimal amount of time covers the setup of preclinical and clinical tests (Phases 0 up to III), that is, the time to ponder upon and write down the study design, to recruit and select patients, to analyze the results, and so on, let alone to perform the actual wet-lab experiments.

Clinical development time (that is, from Phase I) has steadily increased. For drugs approved in 2005–2006, the average clinical development time was 6.4 years, whereas it increased up to 9.1 years for 2008–2012 drug candidates [3]. This might denote an issue in assessing drug effects and benefits. Conversely, the high failure rate of drug development pipelines, often at late stages of clinical testing, has always been a critical issue [4]. In clinical trials occurred between 1998 and 2008 (in Phases II and III), [5] have reported a failure rate of 54%. Main reasons for failure were the lack of efficacy (57% of the failing drug candidates), and safety concerns (17%). Among safety concerns were increased risk of death or of serious side effects, which were still the main reasons of failure in Phases II and III in 2012 [3], and in 2019 [7]. For drug pipelines starting in 2007–2009, the gap in estimated success rates in 2012 was particularly steep between Phase II (first patient-dose) and Phase III (first clinical trial-dose). This means that Phase II, which is related to drug performance assessment, is particularly discriminatory: only 14% of the drug candidates that reached Phase II, compared to 64% of the drug pipelines reaching Phase III, were eventually marketed [7]. This can still be observed for drug pipelines starting in 2015–2017 [6]. 25% of the drug candidates that reached Phase II, compared to 62% of the drug pipelines reaching Phase III, were approved (estimation made in 2019).

**Fig. 2:**
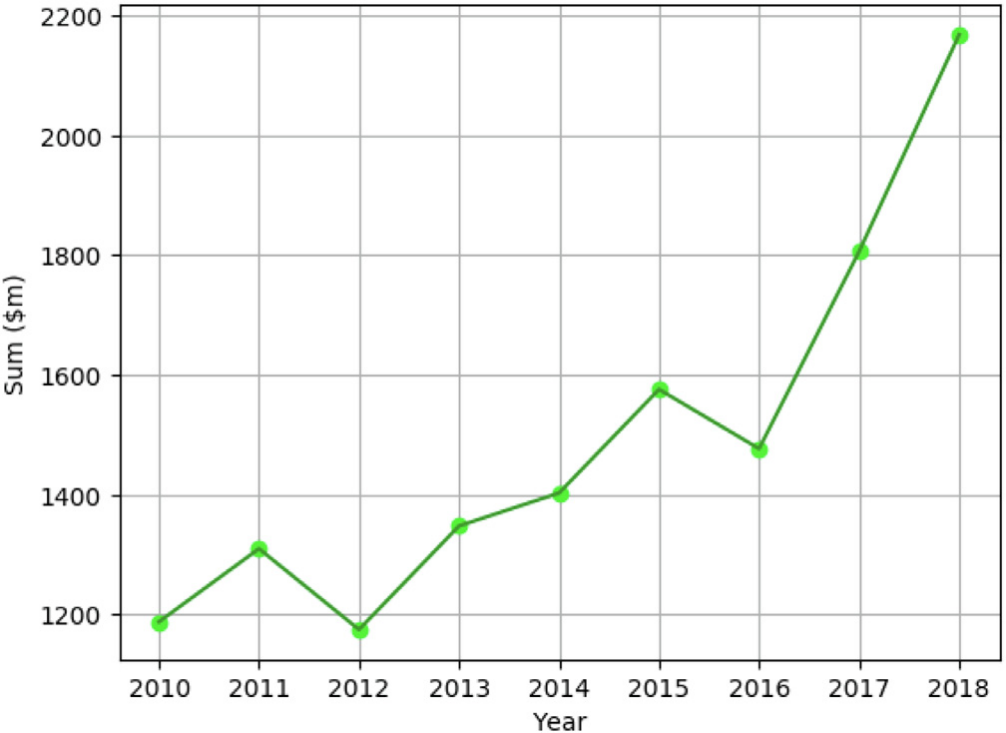
Evolution of average development cost in a cohort of 12 major phamaceutical labs, in millions of dollars, between 2010 and 2018 [10].

Meanwhile, total capitalized expected cost of drug development was estimated at $868 million for approved drugs in 2006, with an average clinical development cost of $487 million, according to the public Pharma projects database [9]. There are large variations due to drug type ($479 million for a HIV drug, compared to $936 million for a rheumatoid arthritis drug) [8]. The recent study of a cohort of 12 large pharmaceutical labs led by [10] shows that total development cost per approved drug has skyrocketed from 2016 to 2018 (from $1,477 to $2,168 million), and almost increased two-fold in eight years (from 2010 to 2018) (see Fig. 2). It is worth noticing that, in the meantime, the number of discovered molecules that have reached the late clinical test stage for this cohort dropped by 22% [10]. Moreover, clinical trials pose a barrier to rapid drug development. The cost and time involved in patient recruitment has been increasing. There is a high failure rate and consequent financial loss in product development. Drug efficiency assessment might not be carried to term because of prematurely-stopped clinical testing, due to the lack of funding [11]. These factors contribute to the decrease in approved drugs.

In a nutshell, all these figures show an expensive, time-consuming, and frustrating R&D context. Although efforts have been made in order to tackle these issues (as shown by the 2019 figures of success rates between Phases II and III), there is still room for improvement in terms of study planning, as suggested in [11], or designing more insightful preclinical testing [1].

### 1.2 The future of drug development

This context has indeed transformed the pharmaceutical industry in the span of ten years. Even the biggest pharmaceutical companies encounter productivity issues, in terms of number of approved molecules with regard to the number of drug candidates [4]. Although a few political efforts have been made to promote orphan disease research [12], this situation has led the pharmaceutical industry to focus on the most profitable diseases. Between 2017 and 2018, the number of active drug pipelines for cancer therapy has increased by 7.6%, whereas the number of anti-infective drugs has dropped by 9.3% [9]. The most studied diseases in 2018, in terms of number of active drug pipelines, are cancer subtypes (breast, lung), diabetes, and Alzheimer’s disease [9]. This observation raises the issue of finding therapies for rarer, complex diseases, where the limited number of patients might hinder meaningful studies to be carried on; or for tropical diseases, where the drug development cost might be too prohibitive with respect to the estimated selling profits [13].

As highlighted by many articles [14], [15], [16], one rather inexpensive way to improve these numbers might be to automate some important but repetitive data processing and analysis tasks, more especially, through robotics [17] and Machine Learning (ML) methods [18]. Indeed, a lot of blossoming collaborations between Artificial Intelligence (AI) and Machine Learning (ML) companies and pharmaceutical labs, as well as universities and research centres [18], [19], [20], slowly bridge the gap in bioinformatics between applied mathematics, computer sciences and biology. This would allow to accelerate drug development pipelines as they might be computationally, thus automatically, performed and less prone to human-related technical mistakes. Authors of [21] estimate their use would shrink the drug candidate identification phase from a few months to one year. Still, one should remain cautious, and not expect computational methods to solve completely the failure rate problem [22]. Nonetheless, integrating ML methods into drug development pipelines might also decrease drug development cost and time [23], and make therapies more patient-oriented, as the easier integration of multiview data might allow implementation or enhancement of precision medicine techniques [24], [25]. Conversely, systematic methods allow study replication and reusability, and enable standardized, transparent data quality control and sharing [26], and *in silico* identification of promising targets. These methods could also provide quantitative values to assess and compare the efficiency of candidate molecules, before any wet-lab experiment or preclinical test.

### 1.3 Towards an automated search for therapies

In order to recontextualize research in drug development, we suggest reading the introductory part of the following referenced papers [13], [23], [27], [28]. Even though a fully-automated drug development pipeline seems out of reach for now [13], the combined efforts from biology, medicine, bioinformatics, computer science and mathematics communities have been spent on improving each part of the drug development pipeline – for example, drug discovery through high-throughput screening (HTS) of drugs via genomic and transcriptomic data [29], genome-wide association studies (GWAS) to uncover new relevant drug targets [30], and increasing application of generic algorithms from machine learning. The use of these methods makes sense in a context where a large quantity and diversity of curated information is available about drugs and their therapeutic indications, disease/aggravating factor targets, disease pathways, and gene/protein regulatory interactions.

## 2 Sequential learning and recommender systems in machine learning

Machine learning (ML) is a subfield of artificial intelligence (AI) in computer science. Here, a ML algorithm designates any computational method where results from past actions or decisions, or past observations, are used to improve predictions or future decision-making. ML techniques are now extremely popular in drug development (see [13], [27], [31] for recent surveys) as they allow automation of highly-dimensional, noisy biological data analysis.

Many different machine learning tasks have been studied, which fall broadly into three categories. The first one is supervised learning, in which the goal is to predict the label of new observations given a large database of labelled examples. Several supervised learning algorithms have been applied in a biological context, such as Support Vector Machines [32] or (Deep) Neural Networks [33]. The second task is unsupervised learning, and it aims at detecting underlying relationships or patterns in unlabeled data. Dimension reduction methods, like Principal Component Analysis (PCA), fall in this class. But other unsupervised problems are also studied in the context of drug development, such as density estimation, clustering (grouping data) or even collaborative filtering. We shall elaborate on some examples below. The third type of task is sequential learning, where algorithms rely on trial-and-error, and iteratively use external observations in order to find the best decision with respect to the environment they interact with.

A large literature has dwelled on the use of sequential learning algorithms, where an agent, that is, a goal-oriented entity interacting with its environment, must make one choice at a time according to previous observations of the environment (from which the input data originate) they are interacting with. While offline –or batch– methods use batches of data in order to learn, online –or sequential learning– algorithms process one data point at a time (receiving a stream of data), and update their prediction or decision accordingly.

**Fig. 3:**
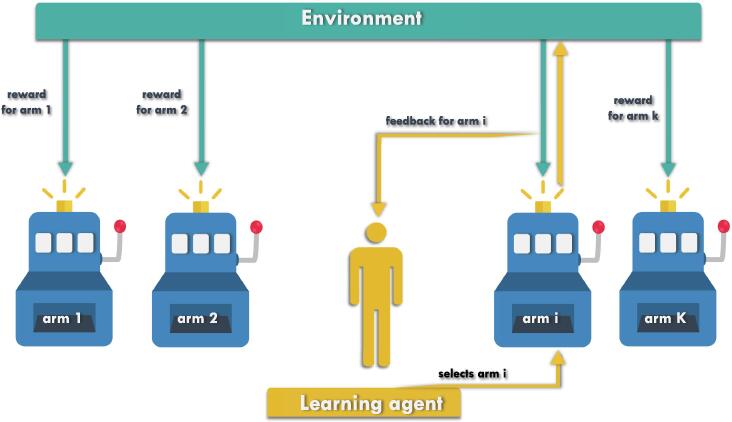
A K-armed bandit, where the learning agent interacts with its environment by sequentially selecting arms, and updating its strategy using the observations it obtains.

Multi-Armed Bandit (MAB) algorithms [34] constitute a popular and versatile family of sequential decision-making algorithms, and were actually motivated by clinical trials, as we shall see. In MABs, a fixed set of actions, called arms, is available. An agent sequentially interacts with the environment by selecting arms, as illustrated in Fig. 3 below. Each arm selection produces some noisy observation, often interpreted as a *reward*. However, the average reward associated with each arm is initially unknown to the agent, and has to be learnt in the process while achieving a certain objective. Typically, this objective could be to discover the most efficient arm(s), that is, the arm(s) with highest average reward, or to maximize the total reward accumulated across iterated arm selections [35]. See the following referenced paper [36] for a comparison between bandit problems.

Recommender systems are closely linked to MABs, as MABs can be used to design sequential recommender systems, see for instance the following referenced examples [37], [38]. Recommender systems actually belong to different families of ML methods, since a recommender system broadly designates an algorithm which aims at predicting rating of a given user which tests a given object. Refer to [39] for a review of the topic. A large part of the literature about recommender systems is motivated by commercial purposes, see for instance [40], [41], [42]. However, we will show that this flexible class of algorithms can actually be applied to solve drug development-related problems.

In the next section, we will review interesting applications of ML in a pseudo-chronological order of appearance in a drug development pipeline –namely, drug discovery, drug repurposing and drug testing– in which established ML algorithms of the three described classes are involved. We will focus on a subset of ML methods, which comprises sequential learning algorithms and recommender systems. Although they are rarely reviewed in a biomedical setting, they have been investigated for tackling drug development-related problems.

## 3 Examples of machine learning applied to drug development

### 3.1 Drug discovery

Drug discovery is usually considered the first stage of a drug development pipeline [43], and is an exploratory step which aims at uncovering putative drug candidates or gene targets, or causal factors, of a given disease or a given chemical compound. A variety of supervised learning methods (for instance, Support Vector Machines and Deep Learning [44], [45], [46], regression methods [47], [48]) and unsupervised learning methods [13] applied to biomedical problems have been thoroughly reviewed in the last decade, with a growing interest in Deep Learning (DL). For further review for DL methods applied to drug discovery, please refer to [33], [49]. Applications may tackle interesting problems in drug discovery: for example, drug candidate identification via molecule docking, in order to predict and preselect interesting drug-target interactions for further research [43]; and protein engineering, that is, *de novo* molecular design of proteins with specific expected binding or motif functions [44].

**Fig. 4:**
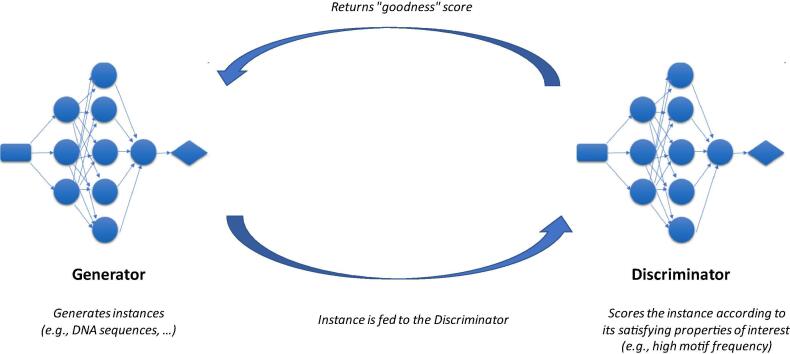
Generative Adversarial Networks for Drug Discovery. A Generative Adversarial Network is a set of two neural networks, the Generator and the Discriminator. These two networks are trained at the same time.

A fairly recent breakthrough in protein design uses generative DL, more precisely, Generative Adversarial Networks (GANs [50]). A GAN is made of two simultaneously trained neural networks (NN) with distinct roles: a Generator, which is trained to sample instances (“generated instances”), and a Discriminator. The goal of the latter is to recognize training instances from generated ones, by assigning them a probability value of the considered instance being sampled from the training set. The objective is to train the Generator to create fake instances that are able to “fool” the Discriminator, that is, which are convincing enough with respect to the underlying “goodness” function represented by the Discriminator. See Fig. 4 which illustrates a GAN. For instance, [51] have applied GANs to generate DNA sequences matching specific DNA motifs, that is, short DNA sequence variants which are associated with a specific function. As a proof-of-concept, highly-rated samples obtained *via* this procedure, exhibiting one or several copies of the desired motif, were shown. Another paper [52] uses, as “predictor“ network, a NN which predicts the probability of the (generated) DNA sequence of coding for an antimicrobial peptide (AMP), and have succeeded in training a GAN which returns 77.08% of the time AMP-coding sequences.

Drug discovery problems have also motivated research in black-box optimization, mostly using Bayesian Optimization (BO), see for instance the following referenced examples [53], [54]. Bayesian Optimization is a field of research for finding global optimum (i.e., either maximizers or minimizers) of a black-box function by sequentially selecting where to evaluate this function. Indeed, the so-called black-box objective function is accessible only through its values at selected points, and might be costly to evaluate. The interest in a sequential strategy is to adaptively choose where to collect information next (which defines a so-called acquisition function). The motivation for viewing some drug discovery tasks as a black-box optimization problem comes from the fact that they usually rely on expensive simulations (at chemical-level), for instance, protein-folding [55], making automatic drug screening and assessment via these operations time-consuming.

The specificity of BO is to choose a prior probability distribution (embedding *a priori* knowledge, simply called prior) on the objective function: e.g., one assumes that the objective function is a sample of a given probability distribution over functions. This prior is then updated after each new function evaluation into a posterior distribution, which in turns guides the selecting of the new point to evaluate. For instance, in [56], the authors apply BO to design gene sequences which maximize transcription and translation rates, from initial sequences. The optimization is performed on feature vectors of fixed length associated with the gene sequences, and the objective function f is the function which, given a gene sequence feature vector, returns the associated transcription and translation rate functions. The prior upon the objective function is a Gaussian Process, classically used in BO [57]. The authors then use as acquisition function the average objective, which will maximize the average of the transcription and translation rates. The feature vector maximizing this acquisition function will then define an optimal gene design rule (for instance, frequencies of amino acid codons) for the maximization of both transcription and translation rates. Since several codons can code for the same amino acid, given a set of sequences coding for a protein of interest, these sequences can be ranked according to the similarity of their corresponding feature vector with the optimal gene design rules that have been derived. The idea is to produce then the protein of interest with lower costs, since the protein production rate is maximized.

Another example of applications of BO to drug discovery is to stimulate the discovery of new chemical compounds [58], that is, finding small molecules that might optimize for a property of interest, while being chemically different from known compounds. This approach might cope with the caveats of previous methods [59].

### 3.2 Drug testing

Once one or several drug candidates are selected, preclinical (Phase 0) and clinical development (Phases I up to III) start. Drug properties, related to body processing of the candidate molecule, should be assessed in early phases: e.g., the ADME properties: absorption, distribution, metabolism and excretion, along with the toxicity levels. Evaluation of their efficacy is performed later during Phases II and III. Automation and development of *in silico* prediction models might save time and money on later testing stages, and subsequent *in vitro* and *in vivo* experiments. Moreover, these methods might come to sometimes replace experiments on animal models, since some agencies start banning animal testing [60]. Such instances of potential *in silico* guiding of wet-lab experiments can be found in [61], [62], [63]; in particular, [62], [63] describe the application of Bayesian multi-armed bandit methods to Phase I studies.

For instance, in [61], a graph-based framework is developed in order to build a prediction model for perturbation experiments, provided time-series expression data and putative gene regulatory interactions between genes of interest. The resulting model can then predict expression levels for the selected set of genes after Knock Out and Over Expression perturbations. This method might help assessing the drug effects on pathways after perturbation of the drug targets. The authors have validated their method on a mouse pluripotency model, with *in vitro* experiments, and report that 60.7% of the predicted phenotypes could be reproduced *in vitro*.

**Fig. 5:**
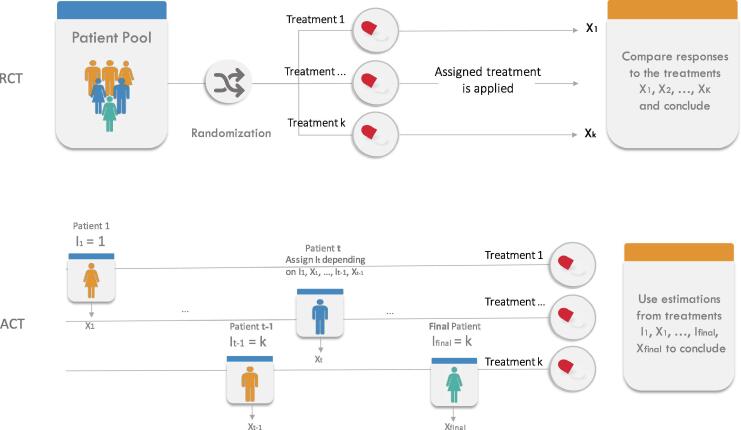
Randomized Clinical Trial (RCT) versus Adaptive Clinical Trial (ACT) for Phase III. A Randomized Clinical Trial (RCT) “randomly” assign patients to treatment arms (ensuring balance of covariates of interest) before testing, whereas an Adapted Clinical Trial sequentially assigns patients to treatment arms according to previous testing results.

Moreover, given the intrinsic sequential nature of a clinical trial, in which patients are given treatments, one (group of) patient(s) after the other, MAB algorithms would be natural candidates to be used in further phases of drug testing. However, nowadays, the motivation for developing new bandit algorithms has entirely shifted to applications to online content optimization, such as sequential recommender systems [42]. Bandit designs appear to have been seldomly used in human clinical trials. Traditional randomized clinical trial (RCT) (where, at the beginning of the trial, each patient from the pool of subjects is randomly assigned to and treated with a random treatment until the end of the clinical trial) have been the gold standard since the 1960’s. Notwithstanding, the past years have witnessed an increased interest in all kind of Adaptive Clinical Trials (ACT), in which the next allocated treatment could be dependent of the outcome of previously allocated treatments. We will now elaborate on the latter. See Fig. 5 for a comparison between RCTs and ACTs.

Seen as a MAB, a Phase III clinical trial proceeds as follows: given K treatments (arms), where each of them has an unknown probability of success p_1_, p_2_, …, p_K_, one of the treatments I_t_ is chosen for the t^th^ patient, and its efficiency (the associated reward value) is subsequently measured. Under the simplest MAB model for a clinical trial, a reward X_t_ = 1 is obtained if the treatment is successful, and X_t_ = 0 if the treatment fails. The allocation is adaptive in that the selection of I_t_ may depend on I_1_, X_1_, I_2_, X_2_, …, I_t-1_, X_t-1_, that is, on the previously given treatments and their observed outcomes. This adaptivity could lead to a smaller sample size to attain a given power, or early stopping for toxicity or futility of a treatment [64], by automatically performing interim analyses [65]. The problem of maximizing rewards in such a bandit model was extensively studied from the 1950’s, either from a frequentist view [66], [67] (where the success probabilities are treated as unknown parameters to be inferred); or from a Bayesian view [68], [69] (where success probabilities are assumed to come from some prior distribution, similarly to Bayesian optimization). Maximizing rewards amounts to maximizing the number of cured patients, which is (arguably) not the purpose of a clinical trial. Yet, the statistical community has also looked at the different problem of finding, as quickly and accurately as possible, the treatment with the largest probability of success. This was studied for example under the name “ranking and selection” [70], [71] and later best arm identification [72], [73]. An interesting take-out from the bandit literature is that the two objectives of treatment identification and curing patients cannot be achieved (optimally) by the same allocation strategy [74].

However, as attractive as the idea of determining the best treatment while treating properly as many patients as possible [75] is, the use of ACTs remains quite rare. This might be due to intrinsic differences between traditional clinical trial methodology and data-dependent allocation, as suggested by [124]. For example, balance between prognostic covariates in each treated group of patients is required for statistical relevance [76]. The use of ACTs might also be hindered by practical reasons in the clinical trial setting, for instance, when one must deal with considerably delayed feedback, as reported by [77]. Nonetheless, these drawbacks might be mitigated by the benefit-risk ratio of the treatment. For life-threatening diseases, an adaptive clinical trial might be a hope for the patients to improve their condition [78], for best empirical treatments, using previous observations, can be assigned to patients.

Despite this initial hostility to ACTs, there has been a recent surge of interest in promoting their actual use. As a notable sign of this evolution, the FDA has updated a draft of guidelines concerning adaptive clinical trials [79], listing in particular several concrete examples of successful adaptive trials. Similarly, [124] presents some examples and promotes some good practice for using ACTs. In the meantime, the authors in [77] have performed multiple simulations illustrating the characteristics of usual bandit algorithms (mostly aimed at maximizing rewards) in terms of allocation and final selection (statistical power), in order to popularize their use.

Among existing bandit algorithms, Bayesian algorithms have achieved a certain popularity. Interestingly, the very first bandit algorithm can be traced back to the work of Thompson in 1933 [35], who suggests randomizing the treatments according to their posterior probability of being optimal. This principle (now sometimes called Thompson Sampling or posterior sampling) was rediscovered around ten years ago in the ML community for its excellent empirical performance in complex models [80]. Interestingly, the use of (variants of) this principle appears to have been proposed as well in different context of drug testing. For example, for phase II trials [81] present a compromise between RCT and Thompson Sampling, while [64] present a proof-of-concept of a phase II trial for the Alzheimer disease that rely on such posterior sampling ideas. More broadly, several examples of successful Bayesian adaptive designs have emerged over the last 20 years, and we refer the reader to [82], [63] for a survey.

Furthermore, research is ongoing in order to tackle for instance the issue of delayed feedback, see for instance [83]. ACTs might allow clinical trials to take explicitly into account inter-patient variability [84], or additional information about the patient [85].

### 3.3 Drug repurposing

The challenges of designing new molecular entities, and testing them through all clinical phases, has generated research interest in a more profitable and efficient technique, called drug repurposing, or drug repositioning. This approach aims at studying already available drugs and chemical compounds to find them new therapeutic indications. This strategy is useful when repurposed drugs have well-documented safety-profiles (that is, side effects and treatments are known), and known mechanism of action.

Different approaches have been used to tackle the drug repurposing problem. For example, some rely on automatic processing of Electronic Health Records (EHR), clinical trial data, and text mining methods to identify correlations between drug molecules and gene or protein targets in literature [86], [87], [88]. However, this approach might be sensitive, but not really specific, since text interpretation is still a hard problem, and the relationship between disease factors and drugs might not be clear. The current state-of-the-art methods seem to have turned to different paradigms of repurposing, see for instance the following reviews for a classification of these different methods [27], [89], [90].

However, most of these methods rely on a rather strong hypothesis, which is that similarity between elements – for instance, chemical composition of drug molecules – implies correlation at therapeutic effect level, or at drug target level. Nonetheless, counter-examples to this hypothesis have been shown to lead to disastrous events: for instance, thalidomide exists as two chiral forms (same chemical composition but having mirrored structures). One of these forms can treat morning sickness; the other form can have teratogen effects [91].

An attempt to quantify more accurately drug effects is signature reversion, also called connectivity mapping, which focuses on expression measurements: given a pathological phenotype (“query signature”) associated with the disease at study, the objective is to identify which treatments are most able to revert this signature. This operation is performed via comparisons of the query signature with so-called drug signatures, that is, vectorized summaries of genewise expression changes due to the considered drugs. This approach has recorded some successes in drug repurposing; see for instance [29] for a comprehensive review of this type of method. However, note that relying only on transcriptomic measurements to understand the mechanism behind a disease might lead to wrong directions when these cannot account for its causal factors. Moreover, drug repurposing procedures that directly use drug signatures extracted from LINCS database [92], either to compute a similarity measure, or to predict treatment effects at transcriptome level, are biased: indeed, the drug signatures that have been computed in LINCS are measured in cancerous or immortalized or pluripotent cells – which (post) transcriptomic regulation might differ from healthy cells.

Furthermore, especially in matrix factorization and some deep learning methods that perform a dimension reduction or, more generally, feature learning on the drug signatures, the learned features often hardly make sense biologically speaking, which prevents easy interpretation of the results and sanity checks.

Another way of solving the drug repurposing problem, as emphasized by [27], is to see it as a recommender system problem, where an agent should “recommend” best available options. Here, the agent should select the most promising drug candidates with respect to the disease or the target at study. Some recent papers have adopted this approach: for instance, in both [89], [93], the authors have designed a graph-based method to predict drug-target or drug-disease interactions. Given a drug, the model predicts a list of fixed length, that contains disease-related targets most likely affected by the chemical compound.

In [89], the algorithm tackles the problem of predicting drug-target interactions (DTI). It relies on a given input bipartite graph of drugs on the one side, and disease-associated gene targets on the other side, which adjacency matrix is denoted A, of size n × m (n is the number of targets, and m the number of considered drugs). An edge connects a drug and a target if and only if the drug targets this gene (meaning, for a pair of nodes (i,j), A(i,j) = 1 if and only if j is a drug targeting gene i, else A(i,j) = 0). In a recommender system point of view, the goal of the algorithm is to determine how probable (high) the considered drug (user) will target (rate) a given gene (object). The authors of [89] compute a weight matrix W, of size n × n, which depends on A and on drug-drug and target-target similarities (importance of each type of similarity might be parametrized): W_ij_ is the coefficient corresponding to the probability that a drug will also target j knowing that it targets gene i. In order to make the inference about the missing edges between drugs and targets, one then computes matrix R = WA.

## 4 Use of data relevant for drug development

In the next section, we give a summary of the fairly new data types that are openly available as of 2019, and that might be useful in regard to drug development challenges, as many datasets can be integrated to training, validation and feature data for the related algorithms. Indeed, what allows ML techniques to really be efficient is publicly available, curated, annotated data. Multiple types of datasets might be relevant with respect to drug development and drug repositioning questions: information about drug candidates, that are, for instance, the chemical structure of the active molecule, disease gene/protein targets, mechanism of action of drugs, but also their documented side effects. One might also be interested in deducing interesting drug candidates by comparing pairs of diseases, of drugs, of protein targets, and applying the principle of “guilt-by-association”. Integrating multi-view data in a drug development method has been shown to increase its accuracy [94], [95], [96]. In this section and in Table 1, we will review currently a few publicly available datasets according to their type which are of interest in a drug development pipeline.

**Table 1:** List of datasets that are relevant for drug development, ordered according to their type. *When provided by the contributors to the database.

Data Type	Description	Databases (name, reference, date of last data update*, URL, size*)	API
Genomic data	(1) Compilation of disease-gene associations; different species are represented in CTD, while the other two databases refer to the human. In CTD, some interactions are manually curated instead of being computationally inferred. OpenTargets and DisGeNET gather data from several curated sources. All of these databases provide a coefficient for each disease-gene association quantifying its corresponding level of evidence.	OpenTargets [97], (2019–11) https://www.opentargets.org/ 27,069 targets × 13,579 diseases	Yes
		Comparative Toxicogenomics Database (CTD) [98], (2019–11) https://ctdbase.org/ Curated: 8,637 × 5816 Inferred: 48,634 × 3168	Yes
		DisGeNET [99], (2019–07) http://www.disgenet.org/ 17,549 targets × 24,166 diseases/traits	Yes
	(2) SNP reporting; COSMIC reports expert manually-curated data.	COSMIC [100], (2019–09) https://cancer.sanger.ac.uk/cosmic 1,207,190 copy number variants 9,197,630 gene expression variants 7,929,832 differentially methylated CpGs 13,099,101 non coding variants	Yes
	(3) Regulatory system (e.g., cis-regulatory modules) data: in CisView, the focus is on the mouse (*Mus musculus*), and data is collected using a TF binding motif analysis on ChiP-seq experiments. It reports several measures of interest, such as conservation scores and quality assessment of the inferred bindings. UK BioBank collects various types of information (genomics, imaging) in a huge anonymous human cohort (around 500,000 people).	CisView [101], (2016–12) https://lgsun.irp.nia.nih.gov/geneindex/cisview.html	No
		UK BioBank [102], (2019–09) https://www.ukbiobank.ac.uk/	Yes
Interaction data	(1) Protein-protein or pathway information; STRING reports PPIs (protein-protein interactions) for thousands of organisms, classified according to their level of evidence: computationally inferred (via functional enrichment analysis), experimentally-proven or extracted from curated databases. A score combining all this information is associated to each PPI. KEGG gathers manually assembled biological (signaling and metabolic) pathways.	STRING database [103], (2019–01) https://string-db.org/ 24,584,628 proteins and 3,123,056,667 interactions	Yes
		KEGG Pathway database [104] (2019–11) https://www.genome.jp/kegg/pathway.html	Yes
	(2) Biological models of gene and pathway interactions; CausalBioNet collects manually curated rat, mouse and human models which are machine readable (encoded into BEL language, convertible into SBML). BioModels lists literature-based (some of them being manually curated) models, and computationally inferred ones, mostly in SBML format.	Causal BioNet [105] http://causalbionet.com/	No
		BioModels [106], (2017–06) https://www.ebi.ac.uk/biomodels/ Manually curated: 831 models Literature-based: 1640 models	Yes
	(3) Drug signatures (genewise expression changes due to treatment) in human immortalized cell lines, from standardized experiments. CMap is a preliminary version of LINCS L1000, and is not supported anymore.	Connectivity Map (CMap) [107] https://portals.broadinstitute.org/cmap/ 1309 compounds × 4 cell lines × 154 concentrations	Yes
		LINCS [92] https://clue.io/lincs 51,423 perturbation types 2570 cell lines 4 doses	Yes
Drug-Disease associations	These databases provide information about disease potential therapeutic targets, along with interacting chemical compounds. PROMISCUOUS reports text-mining (from literature) based associations, however some of the texts are manually curated.	Therapeutic Target Database (TTD) [108], (2019–07) http://bidd.nus.edu.sg/group/cjttd/ 3419 targets × 37316 drugs	No
		PROMISCUOUS [109] http://bioinformatics.charite.de/promiscuous/ 10,208,308 proteins × 25,170 compounds	No
Clinical trials	Repositories of clinical trial settings, status, and results. ClinicalTrials.gov is a large database which mostly collects information about US-located trials (formatted in XML), whereas RepoDB provides visualization and data querying. Clinical trial data is a good source of information for Machine Learning methods, because it lists negative results as well (that is, drugs that failed to prove to be of use in treatment), and potentially the reasons for failure.	RepoDB [110], (2017–07) http://apps.chiragjpgroup.org/repoDB/ 1571 approved drugs × 2051 diseases	No
		ClinicalTrials.gov https://clinicaltrials.gov 323,890 studies	Yes
Chemical & Drug data	(1) Protein-related; automatic annotations.	UniProt [111], (2019–11) https://www.uniprot.org/561,356 proteins (Swiss-Prot dataset)181,787,788 proteins (TrEMBL)	Yes
	(2) Drug-related; comprises approved, withdrawn drugs, as well as tool chemical compounds, and reports their potential indications.	Drug Bank [112], (2019-07) https://www.drugbank.ca/ 13,450 drugs	Yes
	(3) ADMET drug properties (among other types of relevant drug information).	ChEMBL [113], (2018-12) https://www.ebi.ac.uk/chembl/ 1,879,206 compounds × 12,482 targets	Yes

### 4.1 Data types

We mainly focused on datasets which are publicly available online, where the associated data is either easy to download and automatically read, or easy to get access to via an API (Application Programming Interface). This criterion is crucial as it allows data to be easily integrated into a computational method. We required as well that they contain high-quality data – meaning, expert-curated or processed in a relevant way that discards purely correlative assumptions and provides a confidence score – in large quantities, so to avoid having to gather data from different sources which are preprocessed in different, non comparable, ways. Moreover, whenever possible, we also focused on databases which benefited from recent updates (less than one year), in order to ensure that they are still maintained and relevant.

### 4.2 Feature selection

In ML and statistics, feature selection aims at trimming or transforming (raw) input data in order to only feed valuable information to the prediction model. This step should not be considered optional when designing ML algorithms. If not applied to the input data, the algorithm might actually learn artifacts, be biased, or even only learn rubbish. As such, feature selection is of paramount importance in order to ensure study replication and to guarantee that the developed prediction model will be useful [114]. Feature selection allows data denoising and thus reducing the batch effect.

This step can either be manually performed, by carefully selecting biologically-relevant features, and then proceed to test them sequentially and assessing their usefulness; or either automatically, by designing an algorithm which will learn these useful features by itself.

The advantage of the manual way is that good model interpretability usually follows, provided one can quantify from the trained model the strength of the influence of each feature (for instance, it is ensured when using linear regression models). Multiple ML methods exist in order to select a subset of preselected features that satisfies interesting properties, such as being predictive of the expected outcome, and being non redundant. For instance, in a drug development setting, statistical methods, random forests, and gradient boosting algorithms helped to successfully predict therapeutic targets from selected features linking genes and diseases of the Open Targets platform [97], or based on gene expression profiles of the LINCS database [92]. The algorithms used for feature selection are usually classified into three major categories: filter, wrapper and embedded methods, where the latter is a hybrid of the former two. See [115] for a comprehensive introduction to feature selection.

Automatic feature selection algorithms can take advantage of the great flexibility and learning power of Deep Neural Networks, that, provided the raw input data and the expected outcome, create the most discriminant features [116], [117]. For instance, the deepDR approach developed in [116] uses an Auto-Encoder (AE) to generate informative features from heterogenous drug-related data, in order to predict new drug-disease pairs. An AE comprises of two neural networks, one Encoder, and one Decoder. The Encoder will project the raw data onto a latent space of features, such that the Decoder is able to reproduce the expected outcomes given the feature vectors associated with the raw input. However, as in any application of Deep Learning, careful training and regularization of these networks should be performed in order to ensure the relevance of the learned features.

## 5 Perspectives

### 5.1 Seeing the organism as a whole system: integration of system biology-related methods to drug development

The power of systems biology and network-based approaches comes from the analysis of multiple genes in functionally enriched pathway, as opposed to traditional single gene and single target approaches. Integration of system biology-related methods to drug development has been implemented for epilepsy in [118], and has allowed the identification of drug target candidates in a systematic way [29], and of a gene module which global expression is highly anti-correlated to epileptic phenotypes [118]. A whole set of genes (a gene module) can be targeted for treatment instead of screening drugs against a single relevant target. Indeed, papers have emphasized on the importance of small-effect gene in a system biology model, as their belonging to highly interconnected gene regulatory networks (GRNs) implies that any slight perturbation on these genes might impact significantly “core” disease genes [24]. A large and active literature [119] has emerged about the formalization, the building and the validation of such GRNs, along with the identification of gene modules highly correlated with pathological phenotype. As GRNs are assumed to mirror gene activity with regard to other genes’ expression, building them usually require (time-series) expression data. These data can be extracted from databases recording measurements of expression after genewise perturbations – for instance, in [120], which relies on Knock Down gene expression measurements collected in LINCS. Even if not yet described today, it can be expected that future methods will take advantage of the use of a restricted part of a GRN to predict *in silico* the effect of a chemical compound on a set of genes of interest.

### 5.2 Precision, or personalized, medicine

Papers have underlined the variability between patients in terms of disease outcome, drug side effect or even drug action [121] due to genomic variation. Precision medicine aims at tailoring a therapy for a specific patient, by taking into account their transcriptomic profiles, genotype, somatic mutations, etc. [25]. If the integration of multi-view data often allows the prediction model to be more accurate [94], [95], it raises the issue of processing, denoising high-dimensional and heterogeneous data (and how to perform feature selection in this case). Practical issues must also be faced, as the data needed to run the model might not be routinely obtained at any hospital, as noticed in [114].

## 6 Summary and outlook

A few takeaway messages can be highlighted from the vast literature about drug development-related methods.

Firstly, since the 2010’s, there is a widely acknowledged decrease in productivity of the drug development industry (the extent of which may vary according to the disease at study), that is likely due to the increasing complexity of diseases to tackle, and to the high average drug development cost. This observation has led several pharmaceutical groups and labs to be interested in ML techniques, along with robotics, in order to decrease drug development time, and also to share observational data and clinical trial results [26], [122]. Even if these efforts only result in a small decrease in drug failure rate during clinical development, this would be a both financially and scientifically profitable improvement for drug development.

Secondly, data availability and quality are key ingredients for the success of ML methods. There are hundreds of online publicly, curated databases which, provided some scripting efforts, can be integrated modularly to drug development pipelines. The feature selection step, which selects and transforms raw data to generate useful model inputs, has been shown to be of paramount importance in order to understand, and to obtain relevant results from ML applications. A variety of methods is now available in order to tackle this problem, and select valid, discriminatory features for prediction or decision model inputs.

Finally, the use of statistical learning algorithms is not short of challenges and should be handled with care. Nonetheless, the field of research seems ripe enough to be applied in at least semi-automated drug development pipelines; there is a growing number of papers published on both relevant data processing, and on algorithms applied for biomedical purposes. Subtle and powerful ML generic algorithms, such as refined DL architectures and sequential algorithms (that have been proven to be useful in a number of non-biology related fields of research), are becoming more and more prominent in biomedical research. They have been applied, with some success, to inherently complex problems, for instance, for adapted clinical trials, *in vitro* experiment prediction or guiding, or *de novo* protein design. Standardization, systematic validation and comparison of drug development methods on independent datasets are anticipated in the future [123].

In the light of new challenging problems, such as designing algorithms generating targeted recommendations for precision medicine, and modelling drug responses as outputs of a much larger system than a handful of genes, modern ML methods might be a useful tool to further enhance drug development.

## Declaration of Competing Interest

The authors declare that they have no known competing financial interests or personal relationships that could have appeared to influence the work reported in this paper.
